# Elements of Human Anatomy: General, Descriptive and Practical

**Published:** 1854-03

**Authors:** 


### Art. III—ELEMENTS OF HUMAN ANATOMY: GENERAL, DESCRIPTIVE AND PRACTICAL.

By T. G. Richardson, M. D., *Demonstrator of Anatomy in the Medical Department of the University of Louisville, and one of the attendtng Surgeons at the Louisville Marine Hospital;* 8vo pp. xx. 734. Philadelphia: Lippincott, Grambo, & Co.

"What a piece of work is a man! How noble in reason! how infinite in faculty! in form and moving how express and admirable! in action how like an angel! in apprehension how like a God! the beauty of the world! the paragon of animals."

We deem it but an act of justice to the author of the present work that a more extended notice of it should be given than the one which appeared in the January number of this Journal. Owing to the late period at which the work was received a mere bibliographical notice was all that could, under the circumstances, be attempted. We resume our task so much the more willingly for two reasons; first, because the production is one strictly Western in its origin, and secondly, because it is the offspring of a young man born and reared in our midst, and educated exclusively in a Western University, without the *prestige* of Eastern study or foreign travel. In making these remarks, it is not intended in the most remote degree to draw any distinction between Western and Eastern Authors, or to institute any comparison between Western and Eastern Medicine, theoretically or practically considered. Whatever is good, valuable, or reliable, is worthy of our confidence and regard from whatever source it may emanate. The West belongs equally to the United States as the East, the North and the South; they are all parts, God be thanked, of one integral whole, and hence whatever they may contribute, whether in literature, the arts, or the sciences, should be considered as offsprings, not of a sectional, but of a national character. Whenever medical men become politicians, and exert their talents and their influence for sectional purposes and not for the general weal, they disgrace their profession, and prove recreant to its best and dearest interests. Many a good work has been condemned both in this country and in Europe, simply because it did not happen to have had the good fortune to be *born*, although it might have been *ushered* into existence, in a particular city. "Can there any good thing come out of Nazareth." The hand of Christian, Jew and Gentile is at once raised against it. Our motto, like that of our great and immortal Statesman, should be, "The union first, the union last, the union one and indivisible."

Many who read this article will doubtless be disposed to ask, what necessity is there for another work on human anatomy? Are our libraries not already full, even to repletion, with treatises of this description? Are we not in possession of the elaborate systems of Portal, Bichat, Boyer, Meckel, Cloquet, Hildenbrandt, Cruveilhier and Quain, to say nothing of the less pretending volumes of Blandin, Monro, Harrison, Maygrier, Lauth, Wilson, Ellis, Ledwich, and of our own countrymen, Wistar, Horner, and Morton? In a word, what need is there of another text-book, since our pupils have already a much greater number of such works than they can profitably read? We do not deny the right of any one to institute such inquiries; for in this enlightened age and in this republican country people do very much as they please, and it is, therefore, very natural, as well, indeed, as very proper, that they should think and judge for themselves upon all matters pertaining to, or affecting, their interests. But we would respectfully ask, by way of offset against such inquiries, whether the above list, which hardly embraces a tithe of the works on the subject, does not refer, in great measure, to foreign works; works which, on account of the language in which they are written, are either inaccessible to the American student, or which, if attainable, are so in consequence of translations and reprints? Our own country, strange as it may seem, has furnished but few treatises on this branch of medical science, and with these the present performance will, to say the least, bear a most fovorable comparison. We have labored long enough, God knows, under the yoke of Great Britain; the want of an international copy-right law, and the indiscriminate re-publication of foreign works, in all the departments of medicine, have well nigh destroyed our national individuality. While the English language, from its universal diffusion in this country has been an inestimable blessing to the people, by furnishing them from abroad the amplest supply of intellectual nutriment, at a rate so cheap as to bring it within the reach of all, even the most poor and humble, it must be perfectly evident, to every reflecting mind, that the circumstance that all our works are written in this language has been, and will continue to be for a long time to come, a great impediment to the establishment of a national medical literature. In this respect it is a great misfortune that we have no language of our own, even though it were no better than the Chickasaw, Choctaw, or Hottentot; forein works then could reach us only in the form of translations, and many an English book which now rejoices in a reprint in the hands of an avaricious bibliopolist would fall still-born from the press. The fact no longer admits of doubt that many of the current English works of the present day are much more widely circulated in this country than in Great Britain. Take for example, Mr. Ferguson's Practical Surgery, which has passed through at least one edition more in the United States than in England, and which has probably four readers in this country for one at home. The same is true of many other publications. With such books

American works cannot possibly compete. There being no international copyright law, the foreign works can be furnished at nearly half the cost of the native; and, when we add to this circumstance the venal puffs with which these reprints are usually ushered before the American profession, it is not difficult to perceive which work, all other things being equal, will gain the victory.

A second reason why we have no national medical literature, is the fact that many of the English reprints are ushered before our profession under the sanction of the names of American physicians. Formerly there was some excuse for this. In the infancy of our country there were few physicians on this side of the Atlantic competent to compose works; their opportunities for observation were comparatively limited, and few had time or inclination, amid the care and annoyances attendant upon the formation of our medical and charitable institutions, to devote themselves to authorship. But the times have changed. These obstacles have been removed, and the world can no longer accuse us either of ignorance or idleness. Indifferent we may be, but not if we are imbued with a true spirit of patriotism and a genuine love and regard for our profession. It used to be, and probably still is, an easy matter for Eastern publishers to hire men to become sponsors for foreign works, and to lend them, for the paltry sum of fifty or a hundred dollars, their names and their influence. Thus endorsed, they often acquire, like spurious coin, an extensive circulation, far greater, in fact, than they obtained at home, or than their intrinsic merits justified.

Another evil which greatly operates to our disadvantage, and the extent of which can be fully apreciated only by those who are thoroughly acquainted with its nature, is the venal character of a portion of our medical press. Every publisher in the country knows that he has only to send a copy of his reprint to the editors of our medical journals, to obtain from these *guardians* of the profession a flaming puff, or a fulsome notice of the English, Scotch, or Irish emigrant. For the sake of increasing their libraries, at a cost *apparently* so cheap, few of these gentlemen hesitate to sacrifice their personal independence and the true interests of our domestic medical literature. The effect of such a course is to give undue weight and importance to foreign works, and to induce the belief, both in and out of the profession, that we are incompetent to supply our own wants by our own resources. That there are noble exceptions to this statement is unquestionable, but they are mere exceptions, not the rule. They are like angels visits, few and far between. We do not deny to foreign works the right to be heard; they should be heard, and be heard deliberately in the spirit of true and candid criticism; their claims to public confidence should be freely canvassed and honestly presented; all that we protest against is the indiscriminate and wholesale system of puffing, so characteristic of the medical press of this country. Editors guilty of the conduct here alluded to, deserve to be branded as the Arnolds of our profession; for they would not scruple to betray our best interests, and barter away our birth-right for a few shekels of silver.

Fourth, and lastly, for we have not time to dwell upon this topic, the establishment of a national medical literature and the success of American authors, is greatly impeded by the fact, that many, if not most, of our medical schools recommend to their pupils foreign works as text-books. That this is the case the annual announcements of our institutions abundantly attest. To refer merely to what exists at home, we find that in the University of Louisville the following foreign works are embraced in the list of those recommended as text-books to the different courses of lectures: WILSON's and QUAIN's Anatomy, CARPENTER's and KIRKE's Physiology, LISTON's Surgery, CHAILLIE's Midwifery, ASHWELL on the Diseases of Females PERIERA's Materia Medica, WATSON's and STOKES' Practice. Of the works of HORNER, MORTON, DUNGLISON, OLIVER, EBERLE, BECK, GIBSON, and many other excellent American authors, no mention is made in connection with the subject. But this practice is not peculiar to Louisville; it obtains, to a greater or less extent, in every school of this country. The circumstances which induce this state of things could, if time permitted, be very easily traced to their real causes, the disclosure of which would not, it is believed, reflect the greatest credit upon our medical schools.

We have incidentally glanced at a few of the principal causes which, in our judgment, are vitally concerned in retarding and obstructing the development of our national medical literature and the success of our native medical writers. It cannot be denied that a good book will always find readers and patrons, but it is equally certain that its merits are often overlooked in this great sea of foreign reprints. Not the least misfortune arising out of this state of things is, that men, in every respect competent to write, are afraid to become authors, well knowing that a copyright work will stand a poor chance by the side of one on the same subject unencumbered in this manner. At some future time we intend to revert to this subject, and discuss it at some length. For the present, we will only add that it is one of grave importance, demanding the serious consideration of every well disposed American physician.

We have stated that our country has produced but few works on anatomy. The first treatise of the kind ever published in the United States, was the "System of Anatomy" by DR. CASPAR WISTAR, in 1814. Considering the opportunities and the high position of the author, the work was one of a very meagre character, altogether in arrear of the existing state of the science in Europe, which had furnished, years previously, the great and immortal treatises of PORTAL, SABATIER, BICHAT, BOYER, MAYER, SOMMERING, and other writers. It was, in fact, for the most part, nothing but a gross compilation from the monographs of JUNES, MONRO, BELL, and other English anatomists. The first volume, as the author himself acknowledged in his preface, consisted almost *verbatim et literatim* of this kind of material, requiring hardly the use even of the scissors, much less of the pen. The second volume, treating of the viscera, bore at least the impress of originality, but the descriptions, although in the main sufficiently accurate, were exceedingly imperfect, and, compared with those of the writers above named, eminently tame and *formulated.* When his work appeared, DR. WISTAR had been for many years a Professor in the University of Pennsylvania, an institution which could then already boast of an existence of nearly half a century. When we reflect upon these facts—the numerous valuable works that had been published in Europe, and the position of DR. WISTAR as the most distinguished anatomical teacher of his age in this country, we cannot concede to him any of the qualities which should characterize an author. His work evinces neither judgment, industry, erudition, nor even good taste. In the latter, especially, he showed himself to be perfectly wanting. Had he possessed good taste and even a moderate amount of industry he would never have borrowed, as he did, one half of his "system" from British writers, and thus afforded occasion for the scoffs and sneers of foreign reviewers. DR. WISTAR enjoyed a most enviable reputation as a teacher on anatomy, and it cannot be doubted that, had he possessed a proper share of industry, he might have produced a great national work. His language is always well chosen, his style concise yet clear, and his descriptions generally accurate. But we have no desire to be capricious and ill-natured; DR. WISTAR was an excellent anatomist, if not a great author, and, with all the imperfections of his work, we should be grateful to him for having produced it. It was the first effort to *naturalize* anatomical science in the United States; and, regarding it in this point of view, it behooves us to make due allowance for its many sins of omission and commission. With the many valuable additions that have been made to it, since the death of the lamented author in 1818, by DR. HORNER and DR. PANCOAST, who have been successively its editors, the "System of Anatomy" by CASPAR WISTAR is destined to retain, for many years to come, a place in the affection and regard of every American student.

The next work on anatomy was from the pen of DR. HORNER, for several years the assistant of DR. WISTAR and DR. PHYSICK and for nearly a quarter of a century the Professor of Anatomy in the University of Pennsylvania. Appearing in 1826, in two octavo volumes, under the title of "Special and General Anatomy," it had passed through the eighth edition two years before the author's death in the winter of 1853. As it now presents itself, it may be justly regarded as a most able and systematic treatise, thoroughly representing the existing state of the science, creditable alike to our age and country, and reflecting the highest credit upon the school with which DR. HORNER was so long and so usefully associated. In its general scope and character, it is a vast improvement upon its predecessor, and, notwithstanding the inelegancies of its style, will compare favorably with the very best foreign works on the same subject. Indeed, with the exception of the great treatise of CRUVIELHIER, the elaborate system of HILDENBRANDT, and the excellent production of QUAIN and SHARPE, there is not, so far as our knowledge extends, a more valuable and excellent work on anatomy in any language. DR. HORNER was a most zealous and laborious man, with a mind thoroughly imbued with a love of the science which he so ardently and so successfully cultivated for upwards of a third of a century. Indeed, taken all in all, he was one of the most able and accomplished anatomists of the age, and the only great anatomist that our country has yet produced. We do not speak of him here, of course, as a lecturer; in this respect he had many superiors; we refer altogether to his thorough knowledge of the subject, in all its relations, general, descriptive and surgical, to say nothing of the microscopical and pathological departments, in which his information was eminently respectable, if not profound,—to his extraordinary skill as a dissector, and to his zeal and untiring devotion to the cause of anatomical pursuits. Comparative anatomy we believe, never specially engaged his attention, and this is the only branch of the subject which he has not illustrated by his labors. With talents of no high order and many early educational defiencies, he accomplished results of which any man, however ambitious, might justly be proud. If he has not produced the ablest work on human anatomy that has ever been written, he has furnished one which reflects great credit upon our country, and which will serve to transmit his name honorably to posterity along with those of WINSLOW, BOYER, MECKEL, CRUVIELHIER, HILDENBRANDT, and other cultivators of the science.

In 1849 appeared the work of Dr. SAMUEL GEORGE MORTON, whose ethnological writings, especially his elaborate treatises on the "Crania Americana," and "Crania Ægyptiaca," have earned for him a world-wide reputation, and conferred upon him an enviable immortality. The title of the work "An Illustrated System of Human Anatomy, special, general and microscopic," and its motto, "*Oculis subjecta fidelibus*," sufficiently express its nature and object. The book, in its mechanical execution, is one of the most beautiful that has ever been published; the engravings, amounting to nearly four hundred, are executed in the highest style of the art known in this country, and the paper and typography are altogether of a superior quality, nearly equal, in fact, to those of the best English works on medicine. But here the merits of the performance cease. As a literary and scientific production it has nothing in particular to recommend it. In both these respects, indeed, it is inferior to many of the treatises previously in use, and accessible to American pupils. It is difficult to conceive what motive could have actuated the distinguished author in spending his time upon a work which, in no event, could enhance his reputation, or fill a void in the anatomical literature of the country. Following the beaten track, he has introduced no new arrangements in his topics, nor added one solitary fact to our pre-existing knowledge in any of the departments of anatomical science of which he treats. With the dissecting-room, it may be presumed, he has not much familiarity, and with the microscope not much experience. When men have acquired great proficiency and great reputation in one particular branch of science, it behoves them to pause and reflect before they undertake the composition of elementary works. When Dr. MORTON set about this task he had reached the zenith of his renown as an ethnologist, and a period of his life, which should both have admonished him to refrain from an enterprise which any youthful aspirant after fame, with a moderate share of anatomical knowledge, might have executed quite as well as he. Nevertheless, as we have already said in regard to WISTAR's work, we should not undervalue the "Illustrated System" of Dr. MORTON, since, whatever may be its deficiencies, it is calculated rather to add to, than detract, from the dignity and importance of our national literature, a subject in which, as has been already stated, every true-hearted American must feel the deepest interest.

Such was the state of our anatomical literature at the close of the first half of the present century. It will be conceded, even if we include in the enumeration Dr. GOODMAN's Anatomical Investigations, Dr. HORNER's United States Dissector, and Dr. SMITH's Anatomical Atlas, that our stock was comparatively, a meagre one. This circumstance is not a little surprising when we take into consideration the great number of medical schools in this country, and the extraordinary advantages afforded in many sections of the United States for the prosecution of practical anatomy. During the same period, France, Germany, and England have each furnished numerous and important works on the subject, eminently creditable to their authors and to the scientific character of the nations to which they respectively belong. The commencement of the present year has supplied us, almost simultaneously, with two new works on this department of medicine. Of these, one is from the pen of Dr. W. R. HANDY, Professor of Anatomy and Physiology in the Baltimore College of Dental Surgery, and the other from the pen of Dr. T. G. RICHARDSON, Demonstrator of Anatomy in the University of Louisville. These two works, added to those of WISTAR, HORNER and MORTON, give the sum total of five native treatises on the subject; a result astonishingly small when we consider the number of works that have been published in some of the other branches of medical science.

The work of Dr. HANDY, of which the mere mention must suffice, is entitled, "A Text Book of Anatomy and Guide in Dissection," and was professedly written for the use of students of medicine and dental surgery. Upwards of one hundred pages are devoted to the description of the mouth, gums, and teeth, and this circumstance, in the opinion of the author, if not also of the reader, constitutes the great and distinguishing feature of the work, which, in point of mechanical execution is altogether inferior to its cotemporary.

It is not our design to present an elaborate account of the work of Dr. RICHARDSON; all that we shall attempt to do is to delineate its general scope and character. It is in fact, itself an analysis, and to analyze an analysis would be an absurdity.

The work is divided into three parts, the first being devoted to general anatomy, the second to the bones and joints, and the third to the dissection and study of the soft parts. The subject of general anatomy occupies about eighty pages, in which, limited as the space is, is discussed every thing that is really important to be known by the medical student. Until recently the study of the animal tissues was, unfortunately, too much neglected, both in the schools of the United States and of Great Britain, and there is reason to believe that it receives, even at the present day, too little attention. One cause, perhaps, of this is the want of a good text book. For a long time, in this country as well as in England, the only work to which the student could refer for information on this branch of anatomy, was the great treatise of XAVIER BICHAT, translated by Dr. GEORGE HAYWARD, of Boston, and published in that city in 1822, in three octavo volumes. "Before the time of BICHET," as Dr. RICHARDSON justly observes, "no successful effort had been made towards a correct analysis of the body into certain distinct 'textures,' or 'tissues,' which are recognized by the unaided senses, and distributed throughout the different parts and organs. This illustrious Frenchman indicated, and followed as far as his means of study and his too short life permitted, this new direction of scientific anatomy." The work of BICHAT was the first attempt at a systematic arrangement of the tissues, according to their affinities and intimate relations, and the important part which they play in the construction of the different organs and systems of organs of the animal frame. Prior to his time all was darkness and confusion. BORDEU, towards the latter part of the last century had, it is true, written a treatise upon what he improperly called the mucous tissue; but, although his account was in the main suffiently correct, many errors necessarily crept into the work, and the principal good which resulted from the publication was, that it served as a guide board to BICHAT, in pointing out to him the path which he was destined afterwards to tread and pursue with so much credit to himself and advantage to science and humanity. The work which he published at the dawn of the present century, as the result of his labors, was one of vast toil and profound originality, and will, in whatever light it may be viewed, remain as an enduring monument of his genius, his research, and his indomitable industry. It comprises the germs of thousands of discoveries that have since been achieved in the various departments of medicine, especially in physiology and pathology, and is, beyond all doubt, by far the most precious contribution to our science that has ever been made by any individual. The discovery of the circulation of the blood by HARVEY was, in comparison with the revelations of BICHAT in general anatomy, a mere sun-beam as compared with the sun itself. BICHAT was emphatically the BACON of medicine. In him, more than in any other physician before or since his time, were happily concentrated vast genius, profound philosophy, and vast industry, with a spirit of generalization, of which, until then, the medical world knew no parallel. It seems as if Providence had designed him for a special mission, to enlighten and benefit his profession, and that, having accomplished the objects of that mission, he should be prematurely summoned from his earthly toils to join, in a brighter and a better world, the master spirits that had preceded him thither. At the early age of thirty-two nothing remained of this great man, save his image, to his friends, his pupils, and his admirers; and his great and imperishable works to his profession and to posterity. Bichat is the father of general anatomy.

In 1828 appeared the excellent "Manual of General Anatomy," of Bayle and Hollard, in an English translation by Dr. Gross, then of Philadelphia. Soon after this Dr. Togno, also of Philadelphia, published a translation of Beclard's "Elements of General Anatomy." These two works exercised an important influence in directing the taste of our pupils in regard to the study of the tissues, and they continued to be, for a long time, the text-books of our principal schools. Dr. Craigie's well-known work on "General and Pathological Anatomy" was issued at Edinburgh in 1828, and the following year appeared, at London, Mr. Grainger's "Elements of General Anatomy," at that time, and for a considerable period afterwards, the only original treatises on these subjects in the English language!

The following extracts will serve to convey an idea of the author's classification of the tissues:—

"Bichat and his immediate followers, however, admitted as distinct tissues only the solid parts of the body, which in reference to their distribution he arranged into classes, or 'systems,' such as the 'muscular system,' &c. But modern anatomists have most clearly demonstrated that many of the fluids may justly claim a similar designation.

"The number of the solid tissues has been variously estimated from seven to twenty. The following table, however, in which only fifteen are enumerated, will be found to answer all ordinary purposes. The old, and most easily remembered classification, into 'general' and 'partial' tissues, *i. e.* tissues that are found in every part of the body, and those that exist only in certain localities, is here adopted:—

General Tissues.
1. Areolar or cellular tissue.
2. Vascular tissue, comprising blood-vessels, lymphatics, and lacteals.
3. Nervoue tissue.

PARTIAL TISSUES.
4. Epithelial tissue.
5. Pigmentary tissue.
6. Adipose tissue.
7. Muscular tissue.
8. Fibrous tissue.
9. Yellow-elastic tissue.
10. Cartilaginous tissue, including fibro-cartilage.
11. Osseous tissue.
12. Cutaneous tissue.
13. Mucous membrane.
14. Serous membrane.
15. Glandular tissue."

Having given an account of the physical, chemical, and vital properties of the tissues, together with their development and growth from cells, the author proceeds to speak of the individual tissues, commencing with the areolar, cellular, or filamentous. Each of these subjects receives due attention, and is illustrated by beautiful figures, calculated to convey to the mind of the student a just idea of the nature of the object to which they relate.

Part second treats of the bones and joints, and occupies nearly one hundred and fifty pages. It has evidently been composed with much care, and comprises a very full account of these important objects.

Part third, after a brief introduction, in which the student is instructed in regard to the proper method of conducting his investigations in the dead-room, begins with the dissection and description of the abdomen, including an excellent account of the surgical anatomy of inguinal hernia. Under the head of the pelvis and its contents, will be found a very full and lucid description of the perinæum, one of the most important surgical regions of the body; and the anatomy of femoral hernia is very fully considered along with the structures of the inferior extremity. The organs of the senses, the thoracic, abdominal, and pelvic viscera, the blood-vessels, especially the arteries, and the nerves, are all described with much care and at great length. Finally, the work is accompanied by a table of contents and a most elaborate index, so desirable in all treatises of this kind. The woodcuts, nearly two hundred and seventy in number, are executed in the best style of the art, and add greatly to the intrinsic value of the work. The paper and typography will compare favorably with those of similar treatises in England.

In his account of the appendages of the eye, the author has inadvertently fallen into an error respecting the discovery of the tensor muscle of the tarsus, which he ascribes to the late Professor HORNER, of Philadelphia, by whom it was first introduced to the notice of American, French and English anatomists in 1823. If he will refer to Dr. J. C. ROSENMULLER's excellent "*Hand-buch der Anatomie des Menschlichen Körpers*," the third edition of which was published at Leipzig in 1819, he will perceive that Dr. HORNER was anticipated a number of years by the German author.

In describing the *ocular fascia*, which has been made, of late, to play so conspicuous a part in the diseases of the eye, no mention is made of the fact that that structure was first accurately delineated by a gentleman of this city, several years before it began to attract the attention of English and continental writers, in connexion with the subject of strabismus.

These little matters, however, are circumstances of no moment, and such as might very readily have occurred to any one, however extensively acquainted with the literature of anatomy.

One of the most commendable and interesting features of the present work, is the substitution, where this was practicable, of English for Latin terms. In perusing the works on anatomy, hitherto published in this country and Great Britain, the reader's eye and ear are constantly shocked by the monstrous character of the nomenclature which pervades and disfigures every page of these writings. If we must have Latin names, let us have them by all means, but give them to us in their pure and undiluted state, not intermixed at every line with English. Is it not very absurd, for instance, to speak of the internal mammary vein in one paragraph, and in the one immediately succeeding it of the *vena azygos*, of the femoral artery and of the *arteria profunda*, the common carotid and the *arteria innominata*, the parietal bone and the *os frontis*, and, not to multiply examples, of the semi-lunar valve of the eye and the *musculus levator palpebræ superioris*. In our judgement, nothing can possibly be more barbarous, or more repugnant to good taste, than such a medley. The work of our author, will, we doubt not, go far to correct a practice which, while it is in every respect offensive to good taste and good scholarship, admits of no excuse, because there is really not the slightest necessity for it. We have for many years deprecated this practice in the lecture-room, and in a treatise on anatomy, prepared many years ago, but which has never been completed, we adopted precisely the same method as that set forth in the work under consideration. The author deserves much praise for having set an example, which is sure to find many imitators. In France and Germany a uniform anatomical nomenclature has long been in vogue, as every one acquainted with the anatomical literature of those enlightened countries is aware.

Another important feature of the work, is that it combines general with descriptive anatomy, and descriptive with practical, thus enabling the student to investigate these different departments, without compelling him to purchase separate treatises upon each. With many of our pupils this is a very serious desideratum; for to say nothing of the inconvenience involved in referring to different works on the same branches of science, not a few of them are in straitened circumstances, and can, therefore, ill afford to spend their means in this manner.

Finally, the style of the work is always clear and accurate, and hence the author never fails to make himself perfectly understood. Nothing of the ornate is any where visible, for the reason that any attempt of this kind would have been highly inappropriate, if not ridiculous, in a treatise rigidly scientific and descriptive. The author has executed his task ably, faithfully and systematically. Practically conversant with the dissecting-room, he has written from observations made upon the dead subject, and thus produced a work admirably adapted to the wants of the medical student. "*C'est*," says Mons. H. Cloquet, "*avec le scalpel à la main qu'on doit faire un ouvrage d'anatomie.*"

We have said that the author of this work is a young man. This circumstance, so far from being an objection to the work, constitutes one of its chief claims to public patronage. Who but a young man can fully appreciate the wants of the student of practical anatomy? Who but young men frequent the dissecting room? If the work were one on practical medicine, surgery, or obstetrics, we should naturally expect the author to be an older man, not necessarily, however, because old age does not always bring experience; but in a work of this kind, age alone does not confer knowledge, nor necessarily imply wisdom. It would not be difficult to find in the various walks of literature—philosophy, poetry, jurisprudence, theology, medicine, natural history, and physical science—examples of young men who have produced great and noble works as the fruits of their early observation and industry. To go no further than our own profession, it may be stated that ANDREAS VESALIUS, the great anatomical reformer in the sixteenth century—the man who performed the same service for anatomy that Luther performed for religious and political freedom—published his work, "*De Structura Corporis Humani*," when he was scarcely twenty-five years of age, and RICHARDSAND gave to the world, almost before he had attained his majority, his celebrated "Elements of Physiology," one of the most erudite and beautiful treatises of the kind that have ever appeared in any age or country. It was in view of this circumstance that the French author was induced to insert the following sentence in his preface: "If any one should conceive the present undertaking to be above the capacity of my age, I will say, even at the risk of a paradox, that young men are, perhaps, best fitted to compose elementary works; because the difficulties which they have encountered in their studies, as well as the steps which they have taken to overcome them, are still fresh in their memory."

We do not make these remarks by way of apology for our author, for no apology is necessary when a task has been so well executed; nor do we use the phrase "young man" as synonymous with that of "young physic." With the latter we feel no very particular sympathy. True progress, active, steady, persistent exertion, directed to the accomplishment of a specific object, or to the attainment of some great end, requires not the patronage of any particular name for its full and perfect development. Men are judged, the world over, by the result of their labors, as the tree is by its fruit, and it has yet to be shown that mere idle boasting, mere vaunting expressions, or the adoption of a new-fangled name, has ever added one jot or tittle to the advancement, the dignity, or the influence of the medical profession. If "young physic" has done any thing in this way, we are free to confess our ignorance of the fact.

